# Effects of light and nitrogen availability on photosynthetic efficiency and fatty acid content of three original benthic diatom strains

**DOI:** 10.1371/journal.pone.0224701

**Published:** 2019-11-06

**Authors:** Eva Cointet, Gaëtane Wielgosz-Collin, Gaël Bougaran, Vony Rabesaotra, Olivier Gonçalves, Vona Méléder

**Affiliations:** 1 Université de Nantes, Laboratoire Mer Molécules Santé, Nantes, France; 2 PBA-IFREMER, Nantes, France; 3 Université de Nantes, GEPEA, Saint-Nazaire, Nantes, France; University of Montreal, CANADA

## Abstract

Microalgal biotechnology has gained considerable importance in recent decades. Applications range from simple biomass production for food and animal feed to valuable products for fuel, pharmaceuticals, health, biomolecules and materials relevant to nanotechnology. There are few reports of the exploration of wider microalgae biodiversity in the literature on high value microalgal compounds, however, because it is believed that there is little to be gained in terms of biomass productivity by examining new strains. Still, without diversity, innovation in biotechnology applications is currently limited. Using microalgal diversity is a very promising way to match species and processes for a specific biotechnological application. In this context, three benthic marine diatom strains (*Entomoneis paludosa* NCC18.2, *Nitzschia alexandrina* NCC33, and *Staurosira* sp NCC182) were selected for their lipid production and growth capacities. Using PAM fluorometry and FTIR spectroscopy, this study investigated the impact of nitrogen repletion and depletion as well as light intensity (30, 100, and 400 μmol.photons.m^-2^.s^-1^) on their growth, photosynthetic performance and macromolecular content, with the aim of improving the quality of their lipid composition. Results suggest that under high light and nitrogen limitation, the photosynthetic machinery is negatively impacted, leading cells to reduce their growth and accumulate lipids and/or carbohydrates. However, increasing lipid content under stressful conditions does not increase the production of lipids of interest: PUFA, ARA and EPA production decreases. Culture conditions to optimize production of such fatty acids in these three original strains led to a balance between economic and ecophysiological constraints: low light and no nitrogen limitation led to better photosynthetic capacities associated with energy savings, and hence a more profitable approach.

## Introduction

Production of microalgae is gaining interest for the supply of biofuels, feedstock and for added value compounds; several thousand species of microalgae have now been screened for lipid production, including diatoms [1–8]. Diatoms are described as producers of biologically-active compounds: antibiotics, enzyme inhibitors, pharmacologically active compounds and toxins. The most studied molecules are eicosapentaenoic acid (EPA) and arachidonic acid (ARA), which belong to the polyunsaturated fatty acids (PUFA). Fatty acids such as EPA and ARA are considered pharmacologically important for dietetics and therapeutics. They have been used for prophylactic and therapeutic treatment of chronic inflammations (e.g. rheumatism, skin diseases, and inflammation of the mucosa of the gastrointestinal tract) [9]. They are also believed to have a positive effect on cardio-circulatory diseases, coronary heart diseases, atherosclerosis, hypertension, cholesterol and cancer [10]. Fatty acids like saturated fatty acid (SFA) and monounsaturated fatty acid (MUFA) are associated with triacylglycerols (TAG), which are preferred substrates for biodiesel storage production by transesterification [11]. It is essential to identify suitable strains of microalgae for mass cultivation and improve their lipid content. Previous studies have shown that the quantity and quality of lipids can vary as the result of changes in growth condition i.e., nutrient concentrations [12–17], temperature [18–20] and light intensity [12,13,15,19,21]. It is quite common to encounter oil levels reaching 20–50% of microalgal dry weight, although this varies from species to species, and can reach up to 90% of the dry mass when cells are subject to physiological stress conditions, such as nutrient limitation or photo-oxidative stress, or an unfavorable environment [22]. Furthermore, nutrient stress, e.g., nitrogen deprivation, phosphorus starvation, or iron supplementation can also enhance the lipid content in many microalgae species [23–26]. Nitrogen limitation or deprivation is a strategy widely used to elicit this response. Lipid biosynthesis varies within the different diatom species, their growth stages and the environmental parameters [27,28].

Nutrient availability is of considerable importance to growth and primary production in microalgae. For diatoms, typical nutrient limitation in nature involves nitrogen, phosphorus, and silicate [29]. Diatom cells undergoing nutrient limitation change their cellular composition; nitrogen limitation can lead to a decrease in protein content and a relative increase in carbohydrate and/or lipid storage [30,31]. Nitrogen limitation can also result in a decrease in growth rate and photosynthetic efficiency [29,32–34]. The biochemical changes measured in microalgae are linked to changes in physiological parameters and are known to be species-specific. Photosynthetically active radiation can also affect primary production, protein synthesis and other cellular functions [21,35,36].

In the selection of the most adequate species or strains for bioactive lipid production, many parameters need to be considered, such as the ability of microalgae to grow in a wide range of environmental conditions. In this study, three benthic marine diatom species: *Entomoneis paludosa* NCC18.2, *Nitzschia alexandrina* NCC33, and *Staurosira* sp. NCC182, never previously studied except by Cointet et al. (2019) [6], were selected for their high growth capacity, high lipid productivity and/or capacity to produce interesting molecular compounds. The purpose of this study was to examine and characterize their photosynthetic capacity and enhance their lipid productivity for potential future applications. The effects of different light conditions were tested: 30 μmol photon.m^-2^.s^-1^ (Low Light: LL); 100 μmol photon.m^-2^.s^-1^ (Medium Light: ML) and 400 μmol photons.m^-2^.s^-1^ (High Light: HL) in combination with different nitrogen concentrations: 882 μM (N+) and 88.2 μM (N-). The aim of this study is to determine the effects of changing nitrogen concentration and light conditions on growth rate, photosynthetic capacity and intracellular lipid accumulation and composition. Macromolecular composition in relation to culture conditions was evaluated using Fourier transform infrared spectroscopy (FTIR) and photosynthetic capacity obtained from Pulse Amplitude Modulated (PAM) fluorometry measurements, complemented by pigment analysis. Finally, lipid quality was estimated by GC-MS analysis on transesterified lipid extracts to reveal fatty acid (FA) composition and thus to see whether culture conditions impacted production of lipids of interest such as EPA and ARA.

## Materials and methods

### Diatom cultures and experimental design

The three strains, *Entomoneis paludosa* NCC18.2, *Nitzschia alexandrina* NCC33, and *Staurosira* sp. NCC182 were obtained from the Nantes Culture Collection (NCC). Each strain was grown using artificial seawater medium [37] enriched with F/2 medium major nutrients. This artificial medium made it possible to control the initial amounts of nutrients and other elements (Table 1), thus avoiding the variability of natural seawater composition [37]. Two different media were used in the experiments: N+ and N-, with initial NaNO_3_ quantities of 882 μM and 88.2 μM, respectively (Table 1). Before inoculation, the medium was sterilized by filtration (0.2 μm) to avoid the nutrient precipitation that often occurs with autoclaving. Culture stocks were acclimated and maintained for 5 weeks in 250-mL Erlenflasks filled with 150 mL medium N+ under low (LL), medium (ML), and high (HL) light conditions: 30 100–400 μmol photons m^-2^ s^-1^, respectively. A light-dark cycle was applied (14–10 H), and temperature maintained at 16°C. To prevent pH augmentation due to diatom growth [38], cultures were gently bubbled with continuous sterile filtered air (Sartorius, 0.2 μm PTFE) and aseptic glass delivery tube by air compressor.

**Table 1:** Artificial seawater medium composition.

Solutions		Final concentration (μM)
Anhydrous salts	NaCl	336 × 10^3^
	Na_2_SO_4_	28.8 × 10^3^
	KCl	9.3 × 10^3^
	NaHCO_3_	3.3 × 10^3^
	KBr	840
	H_3_BO_3_	48.5
	NaF	71.5
Hydrous salts	MgCl_2_.6H_2_O	54.6 × 10^3^
	CaCl_2_.2H_2_O	10.5 × 10^3^
	SrCl_2_.6H_2_O	63.8
Major nutrients	NaH_2_PO_4_.H_2_O	36.2
	NaNO_3_	882 (N+) or 88.2 (N-)
	Na_2_SiO_3_.9H_2_O	106
	Trace metal solution (F/2)	1 mL
	Vitamin solution (F/2)	1 mL

At the start of the experiment, fresh medium was inoculated using cells from stock cultures that were centrifuged (5 min at 3500 *g*) and washed with N- artificial seawater medium to avoid salt concentration. The initial concentration of cells was set at 30.000 cells.mL^-1^ in 150 mL sterilized medium for both nitrogen depleted (N-): 88.2 μM, and nitrogen replete (N+): 882 μM (N+) media, and cultures were exposed to the three light conditions: LL, ML and HL. Samples were collected daily for growth rate estimation by cell counting until the cultures reached stationary growth phase. During exponential growth phase, the photosynthetic parameters (maximum PSII quantum efficiency, Fv/Fm and light saturation parameter Ek) were estimated using PAM-fluorometry [39]. Thus, culture volumes necessary to harvest 10^7^ cells were sampled and centrifuged (10 min at 4500 *g*) for biochemical composition determination: pigment composition by spectrophotometry [40] and protein, carbohydrate and lipid composition by HTSXT-FTIR [6]. The total cells in culture were harvested by filtration (0.7 μm) to estimate DW and perform lipid extraction following Bligh and Dyer [41]. Media free of cells were recuperated during the filtrations to then estimate nutrient composition using a DIONEX ion-chromatography [42].

### Nutrients

Before inoculation, 50 mL of fresh medium were sampled. Medium samples were also taken by filtration during the cell harvesting process. In this case, 50 mL of filtered medium were frozen at -80°C for conservation until measurement of nitrogen, phosphate and silica content. Residual phosphate (PO_4_^3-^ μM) and nitrate (NO_3_^-^ μM) concentrations were analyzed according to the method described by Aminot and Kérouel (2007). After being thawed, samples were centrifuged (10 min at 3000 *g*) and colorimetric assays were carried out on the supernatant with an AA3 autoanalyzer (SEAL Analytical^®^), which allows automatic nutrient determination by continuous flow spectrophotometry. Determination of PO_4_^3-^ with this method relies on the reaction of molybdate with antimony to form the phosphomolybdic complex. This complex is then reduced by ascorbic acid to form a blue compound that is measured at λ = 820 nm. Nitrate is initially reduced to NO_2_ using a cadmium column treated with copper in the presence of two reagents (ammonium chloride and sodium hydroxide). Total NO_2_ is then determined by reaction with sulfanilamide to produce a diazo that reacts in turn with N-naphtyl-thlenediamine in an acid medium and gives a pink coloration, assayed at λ = 540 nm.

Residual silica (SiO_3_^2-^ μM) was determined by colorimetric assay according to Hansen & Koroleff (1999) [43], based on the formation of silicomolybdic acid, which was then reduced to produce an intense blue color assayed at 870 nm. Nutrient consumption by cells during growth was calculated as the difference between initial and final concentrations for each major nutrient (-NO_3_^-^, -PO_4_^3-^, -SiO_3_^2-^). Ammonium (NH_4_^+^ μM) was also analyzed at the end of the growth period because it is known to be produced by bacteria and can be utilized by diatoms for growth [44]. This nutrient was measured to estimate its potential use by cells and its possible interaction with nitrogen conditions tested. NH_4_^+^ was analyzed with the colorimetric indophenol blue method adapted to seawater assayed at 630 nm (Koroleff 1970) directly on cell-free media without freezing [45] because ammonium was unstable in the samples meaning they had to be processed in the shortest time possible [46]. Final NH_4_^+^ concentration was used to detect whether production occurred during growth.

#### Growth rate estimation

Daily triplicate samples of 2 mL were fixed with lugol and counted (n ≥ 300) using a Neubauer hemocytometer and an optical microscope (OLYMPUS CH40, ×400). Following Cointet et al. (2019), maximum cell concentration (A expressed as a log) and latency time (λ in days) were determined by fitting growth kinetics data with a Gompertz model using Matlab software (Eq 1):

$$f(x)=A\times e^{-e(\mu max\times \frac{e^{1}}{A}\times (\lambda -x)+1)}$$ (1)

with A: maximum cell concentration in the natural logarithm of the biomass; μmax: Maximum growth rate (day^− 1^); λ: Latency (days).

The relative growth rate (day^-1^) was calculated following Eq 2, where N_t1_ is the number of cells on the first day of the exponential phase (t_1_), N_t2_ is the number of cells at the end of the exponential phase (t_2_), and T (days) is the interval between t_1_ and t_2_.

$$\mu =\frac{1}{T({lnN}_{t2}-{lnN}_{t1})}$$ (2)

#### Determination of photosynthetic parameters

During the exponential growth phase (“mid” sampling), and at the end, when the stationary phase was reached (“end” sampling), 2 mL aliquots of the cultures were sampled for photosynthetic parameter estimations using a Water-PAM fluorometer (cuvette version, Walz GmbH, Effeltrich, Germany). Seven hours after the light was switched on, an aliquot was sampled. It was first dark adapted for 1 hour, and then introduced into the 10 mm quartz glass cuvette of the PAM fluorometer controlled by WinControl-3 software. Measurements were performed on the same amount of Chl *a* and cultures were diluted in N- or N+ artificial sea water medium depending on the treatment. Maximum PSII quantum efficiency (Eq 3) was then measured using a 600 ms saturating pulse (2500 μmol photons.m^-2^.s^-1^):

Where F0 is the minimum fluorescence yield for dark adapted cells, Fm, the maximum fluorescence yield for dark adapted cells during the saturating flash and Fv the variable fluorescence.

Fv/Fm=(Fm-F0)/Fm (3)

To provide detailed information on the overall photosynthetic performance of the microalgae [39], RLCs were constructed using nine 30s incremental irradiance steps (0, 38, 52, 77, 121, 179, 259, 391 and 580 μmol photons m^-2^s^-1^) and calculating relative electron transport rate (rETR, Eq 4) through PSII for each level of actinic light [47]:

$$rETR=(\frac{F^{\prime }m-F}{F^{\prime }m})\times PAR\times 0.5$$ (4)

where PAR was the actinic irradiance (= Photosynthetic Active Radiation from 400 to 700 nm), (F’m-F)/F’m was the effective quantum yield of PSII with F, the fluorescence yield for a given PAR intensity, F’m the maximum fluorescence for a given PAR intensity during the saturating flash and 0.5 was a multiplication factor based on the assumption that 50% of the absorbed quanta are distributed to PSII [47].

rETR value data were fitted using the Eilers and Peeter model [48] in order to obtain the initial slope (α), the light saturation parameter (Ek) and the maximum relative electron transport rate (rETRmax). Ek was derived from rETRmax and α (Eq 5):

Ek=rETRmax/α (5)

#### Pigment composition

To estimate chlorophyll a (Chl a) and the total amount of carotenoid pigments (Car) per cell, 2 mL of culture were sampled and centrifuged at 11200 g. for 5 min at the end of the growth phase. After supernatant removal, pigments were extracted by adding 2 mL of methanol (99.9%) to the pellet. To remove cell debris, methanol suspension was centrifuged for 5 min at 11200 g. Absorbances at 665, 632, and 480 nm were measured by spectrophotometer (SHIMADZU, UV-1900) on the clean supernatant to calculate pigment content, expressed in pg.cell^-1^ following [40]:

$$Chl\;a=\frac{13.26\times A665-2.68\times A632}{Number\;of\;cells}\times 10^{6}$$ (6)

$$Car=\frac{4\times A480}{Number\;of\;cells}\times 10^{6}$$ (7)

### Lipid analyses

#### Fourrier-transform infrared spectroscopy (FTIR)

FTIR spectra acquisition was performed according to recommendations of Coat et al. (2014) [49] adapted by Cointet et al. (2019) [6] for benthic diatom strains. The sample preparation and FTIR device are detailed in Cointet et al. FTIR spectra were recorded in transmission mode on 5 μL dried harvested cells (10^7^ cells.mL^-1^) allowing to quickly obtain their biochemical signatures expressed in terms of total lipids, total proteins and total carbohydrates. Absorbance spectra were collected between 4 000 cm^-1^ and 700 cm^-1^ with 30 scans and averaged. The spectra were analyzed by the integral ratios method [50]. The lipid signature was associated with the ester bond (Eb) signal (~1740 cm^-1^), whereas the carbohydrate signature was associated with the C-O-C signal of the polysaccharides (~1200–900 cm^-1^) [37] and the protein signature associated with the amide II band (~1540 cm^-1^) of the N-H of the amides associated with the proteins. To estimate the relative content of lipids, carbohydrates and proteins, their respective integral area was standardized with the total spectrum area and expressed in arbitrary units (a.u.) as recommended by Cointet et al. (2019) [6] (Eq 8).

$$FTIR=\frac{Peak\;area(s)\;}{Total\;spectra\;area}$$ (8)

Where s = lipids (~1740 cm^-1^) or carbohydrates (~1159 cm^-1^) or amide II (~1540 cm^-1^).

#### Gravimetry

Finally, biomass dry weight was estimated by filtering the remaining algal suspension (47 mm, 0.7 μm pore diameter). Filters with cells were washed using 10 mL of ammonium formiate (68 g.L^-1^) to remove salt, frozen at -80°C, freeze-dried and weighed (DW in mg). Crude lipid extract (CLE in mg) was estimated following Cointet et al. (2019) and lipid content (LC) was estimated in mg.L^-1^. Lipid rate (LR) and lipid productivity (LP) were finally calculated as follows:

$$LR=\frac{CLE\;}{DW}\times 100$$ (9)

With CLE and DW expressed in mg

LP=LC×μ (10)

With LC expressed in mg.L^-1^ and μ in day^-1^

#### Fatty acid composition

Fatty acid composition was determined by direct transesterification. A mixture of 800 μL hydrochloric methanol 7.5% and 100 μL chloroform were added to the CLE and heated to 80°C for 5 H. After the reaction was complete, the samples were cooled to room temperature and mixed with 500 μL hexane, which allowed phase separation. The organic phase, which contained fatty acid methyl ester (FAME), was collected, dried using an anhydrous sodium sulfate salt, filtered and evaporated using nitrogen. FAME was analyzed using gas chromatograph mass spectrometry (GC-MS; Hewlett Packard HP 7890 –GC System / Agilent Technologies, Santa Clara, CA, USA) linked to a mass detector (HP 5975C– 70 eV). The sample was injected (1μL injection volume) into a SLB^™^-5ms column (60 m × 0.25 mm × 0.25 μm). The carrier gas was helium at a flow rate of 1mL.min^-1^. The injector and detector temperatures were set at 250°C and 280°C, respectively, and the temperature column was programmed with a temperature held at 170°C for 4 min and then increasing to 300°C at 3 °C.min^-1^. To identify and quantify the FAME, each FA identification was confirmed by comparing mass spectra and retention data with a library build from previous analyses and commercial standards. Chromatogram peak areas were analyzed and quantified using WSEARCH32 software.

### Data processing

Each treatment (N+, N-, LL, ML and HL) was done in triplicate for each strain. Data were expressed as the mean of each triplicate ± standard deviation (SD). Two-way ANOVAs with a 5% significant level were carried out after checking normality and homogeneity using Shapiro-Wilk and equal variance tests. Tukey’s least significant tests was used to determine which experimental conditions were significantly different. All statistical analyses were carried out using SigmaPlot software.

## Results

### Nutrients

Initial major nutrient concentrations (Table 2) were respected for both media (N+ and N-) and for all species. For *E*. *paludosa* and *N*. *alexandrina*, in N+ conditions, nitrate consumption (NO_3_^-^) was higher under HL and ML than under LL (*p* < 0.001) (Table 3). As expected, under N- conditions, all the NO_3_^-^ available in the medium (~83 μM) was consumed in HL, ML, and LL. For *Staurosira* sp., under N+ conditions, the same amount of nitrogen was consumed in LL, HL, and ML (*p* = 0.30); however, as for the two other species, nitrogen consumption was higher in N+ than in N- (*p* < 0.05).

**Table 2:** Initial major nutrient concentrations at the start of the experiment for the different species and culture media (N+ and N-).

	NO_3_- (μM)		PO_4_^3-^ (μM)		SiO_3_^2-^ (μM)	
	N+	N-	N+	N-	N+	N-
*E*. *paludosa*	828.54	83.56	37.31	36.31	117.08	114.89
*N*. *alexandrina*	868.63	74.53	37.54	37.73	111.04	108.33
*Staurosira* sp.	894.66	81.62	39.59	39.34	117.70	116.87

**Table 3:** Nutrient consumption by the end of the experiment (triplicate mean ± SD) for all species and treatments, with the corresponding two-way ANOVA results (n = 3).

Species	Statistical results	Light	Nitrogen	NO_3_^-^ (μM)	PO_4_^3-^ (μM)	NH_4_^+^ (μM)	SiO_3_^2-^ (μM)
*E*. *paludosa*		LL	N+	285.86 ± 57.79	19.45 ± 2.55	0.08 ± 0.10	116.18 ± 0.86
			N-	83.37 ± 0.04	23.50 ± 0.22	< LOD	114.51 ± 0.12
		ML	N+	430.89 ± 33.12	28.81 ± 0.89	2.71 ± 1.43	116.84 ± 0.15
			N-	83.42 ± 0.04	25.45 ± 0.62	0.64 ± 0.40	114.79 ± 0.18
		HL	N+	458.83 ± 28.47	27.61 ± 2.35	2.89 ± 0.80	116.52 ± 0.61
			N-	82.14 ± 1.58	22.15 ± 0.83	0.50 ± 0.81	114.27 ± 0.55
	Two-way ANOVA						
	Light conditions	P		<0.001***	<0.001***	0.004**	0.245
	N concentration	P		<0.001***	0.049	0.001*	<0.001***
	Interaction	P		<0.001***	<0.001***	0.05	0.598
*N*. *alexandrina*		LL	N+	212.18 ± 40.72	36.46 ± 1.34	< LOD	108.29 ± 4.22
			N-	73.23 ± 1.86	36.69 ± 1.14	< LOD	108.33 ± 0.02
		ML	N+	322.96 ± 63.24	36.73 ± 0.10	2.31 ± 1.34	111.04 ± 0.01
			N-	73.78 ± 0.11	34.60 ± 1.91	< LOD	108.33 ± 0.01
		HL	N+	334.11 ± 57.97	37.09 ± 0.14	1.18±2.04	111.04 ± 0.01
			N-	73.30 ± 1.76	35.51 ± 0.59	< LOD	108.33 ± 0.01
	Two-way ANOVA						
	Light conditions	P		0.034*	0.155	0.176	0.329
	N concentration	P		<0.001***	0.004**	0.029*	0.05
	Interaction	P		0.035*	0.031*	0.176	0.321
*Staurosira* sp.		LL	N+	82.19 ± 14.57	13.91 ± 0.48	0.30 ± 0.53	117.43 ± 0.42
			N-	80.72 ± 0.20	14.33 ± 0.48	< LOD	117.08 ± 0.47
		ML	N+	142.00 ± 54.80	18.22 ± 0.98	< LOD	117.63 ± 0.16
			N-	79.96 ± 0.57	16.59 ± 1.35	< LOD	117.15 ± 0.63
		HL	N+	156.44 ± 83.17	19.89 ± 0.73	0.75 ± 1.30	116.73 ± 0.99
			N-	79.56 ± 2.62	19.32 ± 0.40	0.35 ± 0.61	117.63 ± 0.12
	Two-way ANOVA						
	Light conditions	P		0.30	<0.001***	0.543	0.805
	N concentration	P		0.033*	0.145	0.383	0.931
	Interaction	P		0.28	0.136	0.527	0.095

Significance levels: *** for *p* < 0.001; ** for *p* < 0.01 and * for *p* < 0.05. LOD = Limit of detection.

For *E*. *paludosa*, as for nitrogen, more phosphate was consumed in N+ conditions under HL and ML than under LL (*p* < 0.001). However, the same amount of phosphate was consumed in N+ and N- conditions (*p* = 0.05). For *N*. *alexandrina*, phosphate concentration available in the medium was very low at the end of the experiment (< 5μM) in all tested conditions. These results suggest a phosphate limitation at the end of the experiment in all treatments. However, phosphate consumption was higher under N+ than N- for both HL (*p* < 0.01) and ML conditions (*p* < 0.05). For *Staurosira* sp. less than half of the phosphate concentration available in the medium was consumed. Phosphate consumption decreased with light and was highest under HL whatever the nitrogen conditions (*p* < 0.001). For all species and conditions tested, silica and ammonium were present in trace amounts at the end of the experiment. All the silica available in the medium was consumed by the end of the experiment for each condition and each species. Presence of a low concentration of NH_4_^+^ suggests that a low level of production occurred that did not modify our nitrogen culture conditions.

For *E*. *Paludosa*, NO_3_^-^ and PO_4_^3-^ consumption was impacted by light. Nutrient consumption was more important under HL and ML. For *N*. *alexandrina*, consumption was mainly affected by nitrogen concentration. For *Staurosira* sp., only NO_3_^-^ consumption was affected by nitrogen concentration, consumption was the same whatever the light level.

Differences observed when results were expressed in μm.cells^-1^ were the same for each species and treatment.

### Growth

**Fig 1:**
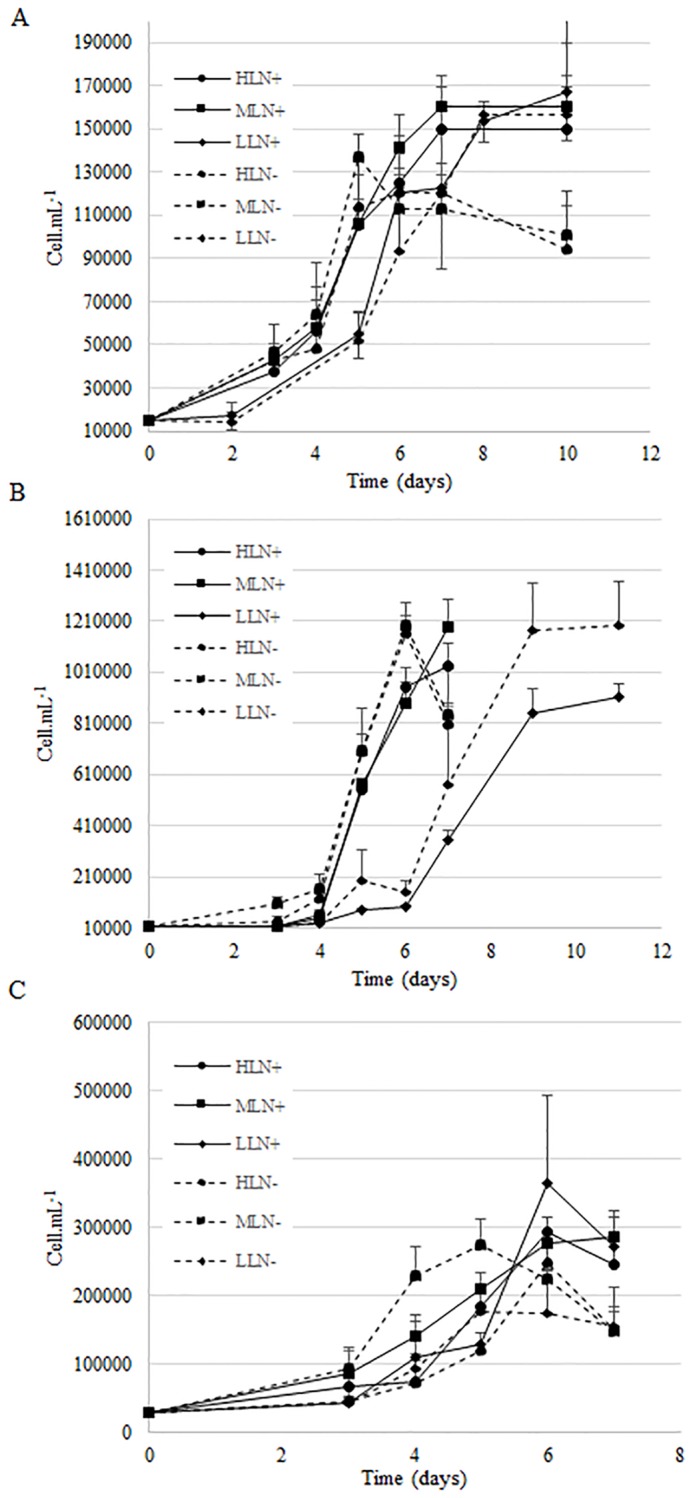
Growth curves expressed in cell.mL^-1^ of *E*. *paludosa* (A), *N*. *alexandrina* (B), and *Staurosira* sp. (C) as a function of time under different light (LL, ML, HL) and nitrogen (N+, N-) conditions.

For *E*. *paludosa*, growth rate (μ) was higher in N+ than N- conditions for ML (*p* < 0.05) and HL (*p* < 0.01) (Table 4). In LL, however, no significant difference was detected between N+ and N- (*p* = 0.77) (Fig 1A). Maximum biomass (A) was the highest under N+ whatever the light level (*p* < 0.01). Latency phase (λ) was impacted by light level (*p* < 0.01), being longer for LL, was not significant affected by nitrogen level (*p* = 0.824). These results can be explained by NO_3_^-^ and PO_4_^3-^ consumption: more nitrogen was consumed in N+ under HL and ML, allowing higher growth rate and biomass, whereas the LL light intensity was insufficient to allow efficient growth (Table 4), inducing lower nutrient consumption (Table 4).

**Table 4:** Growth rate (μ in day^-1^), maximum cell concentration (A in log) and latency time (λ in day) (triplicate mean ± SD) for all species and culture conditions and the corresponding two-way ANOVA results (n = 3).

Species	Statistical Results	Light	Nitrogen	μ (day^-1^)	A (log)	λ (day)
*E*. *paludosa*		LL	N+	0.29 ± 0.06	**2.56 ± 0.12**	**2.84 ± 0.47**
			N-	0.30 ± 0.01	2.26 ± 0.10	**2.80 ± 0.71**
		ML	N+	**0.35 ± 0.01**	**2.48 ± 0.02**	1.45 ± 0.28
			N-	0.30 ± 0.02	2.00 ± 0.11	1.70 ± 0.65
		HL	N+	**0.37 ± 0.01**	**2.61 ± 0.38**	1.65 ± 0.16
			N-	0.28 ± 0.02	2.33 ± 0.15	1.26 ± 0.60
	Two-way ANOVA					
	Light conditions	P		0.090	0.114	<0.001***
	N concentration	P		0.003**	<0.001***	0.824
	Interaction	P		0,020*	0.597	0.582
*N*. *alexandrina*		LL	N+	**0.67 ± 0.02**	4.62 ± 0.09	**4.38 ± 0.07**
			N-	**0.73 ± 0.02**	4.65 ± 0.30	**4.42 ± 0.08**
		ML	N+	1.05 ± 0.10	4.41 ± 0.17	3.45 ± 0.38
			N-	0.83 ± 0.11	4.61 ± 0.25	1.51 ± 0.52
		HL	N+	1.05 ± 0.07	4.16 ± 0.16	3.56 ± 0.09
			N-	1.16 ± 0.13	4.40 ± 0.20	2.65 ± 0.41
	Two-way ANOVA					
	Light conditions	P		<0.001***	0.037*	<0.001***
	N concentration	P		0.694	0.131	<0.001***
	Interaction	P		0.012*	0.646	<0.001***
*Staurosira* sp.		LL	N+	0.69 ± 0.18	**2.81 ± 0.96**	2.65 ± 0.14
			N-	0.61 ± 0.08	1.75 ± 0.13	2.60 ± 0.31
		ML	N+	0.46 ± 0.05	**2.50 ± 0.56**	1.65 ± 0.50
			N-	0.56 ± 0.24	2.08 ± 0.05	2.66 ± 0.16
		HL	N+	0.49 ± 0.12	**2.35 ± 0.19**	2.22 ± 1.51
			N-	0.57 ± 0.04	1.80 ± 0.47	3.37 ± 0.69
	Two-way ANOVA					
	Light conditions	P		0.217	0.674	0.293
	N concentration	P		0.647	0.012*	0.054
	Interaction	P		0.497	0.499	0.296

Significance levels: *** for *p* < 0.001; ** for *p* < 0.01 and * for *p* < 0.05. Remarkable value in bold: for details see text.

For *N*. *alexandrina*, growth rate (μ) increased with light intensity regardless of nitrogen level (*p* < 0.001). There was a significant difference in maximum biomass (log A) among light levels (*p* < 0.05), HL and LL showing the lowest and the highest values, respectively (*p* < 0.05). Conversely, there were no significant differences in growth parameters due to nitrogen conditions (*p* = 0.646), although a drop in cell concentration in N- medium occurred at the end of the growth in HL and ML (Fig 1B). As for *E*. *paludosa*, the latency phase (λ) was longer under LL conditions whatever the nitrogen concentration (*p* < 0.001). These results showed that the impact of light prevailed over the impact of nitrogen in terms of growth. Even if nitrogen consumption was higher under N+, it did not affect the growth. The other hypothesis was that phosphate limitation occurred in N+ and N- conditions. All the available phosphate was consumed, which could have limited growth in the same way, whatever the nitrogen concentration, and explain the drop observed in N- conditions. Under LL, the low growth led limitation to occur later than under HL and ML, due to slower nutrient consumption.

For *Staurosira* sp. there was no significant difference for growth rate between light conditions (*p* = 0.21) and nitrogen concentration (*p* = 0.64). Mean growth rate was 0.56 ± 0.08 day^-1^ for all tested conditions. As for *E*. *paludosa*, maximum biomass (A) was the highest under N+ condition whatever the light levels (*p* < 0.05). Contrary to *E*. *paludosa* and *N*. *alexandrina*, latency phase (λ) was not impacted by culture conditions in this species (*p* = 0.29) and lasted 2.52 ± 0.79 days in all treatments. As for *E*. *paludosa*, more nitrogen was consumed under N+ conditions, explaining the higher biomass obtained. However, no marked trend in growth appeared for *Staurosira* sp, in contrast to *E*. *paludosa*, where growth was more conditioned by N concentration (Fig 1A), and to *N*. *alexandrina*, where growth was more conditioned by light (Fig 1B). For *Staurosira* sp. there was no significant difference in growth except at the end of the growth phase where biomass decreased in N- conditions (Fig 1C).

### Photosynthetic performance

**Fig 2:**
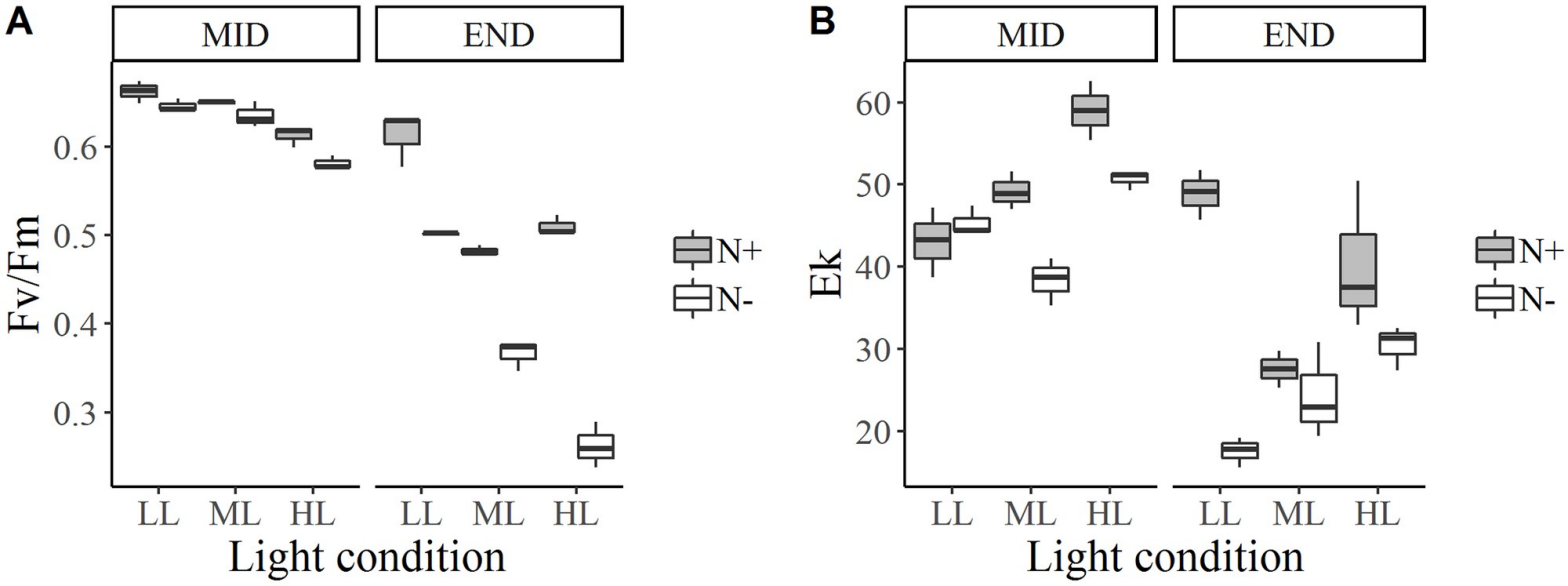
Box and whisker plots for *E*. *paludosa* photosynthetic parameters: Maximum PSII quantum efficiency, Fv/Fm (A) and maximum light saturation parameter, Ek (B) for each culture condition and for two growth stages: Exponential growth phase (mid) and stationary phase (end).

**Fig 3:**
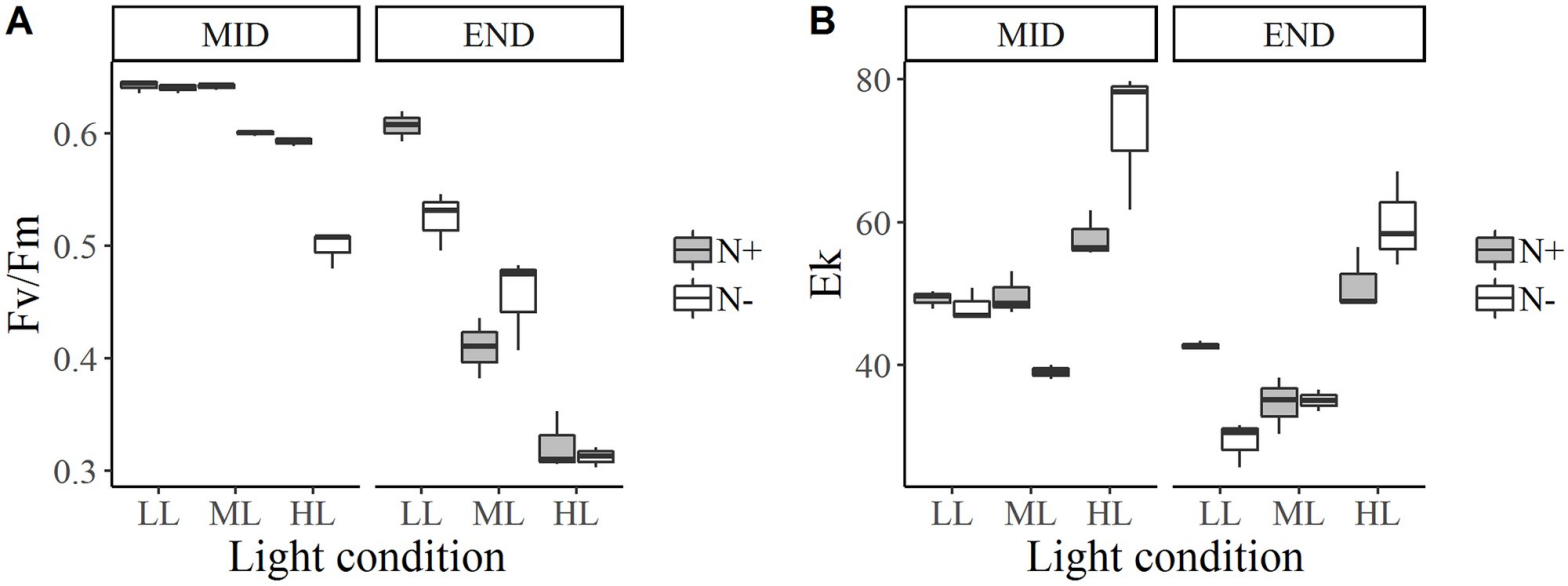
Box and whisker plots for *N*. *alexandrina* photosynthetic parameters: Maximum PSII quantum efficiency, Fv/Fm (A) and maximum light saturation parameter, Ek (B) for each culture condition and for two growth stages: Exponential growth phase (mid-growth) and stationary phase (end of growth).

**Fig 4:**
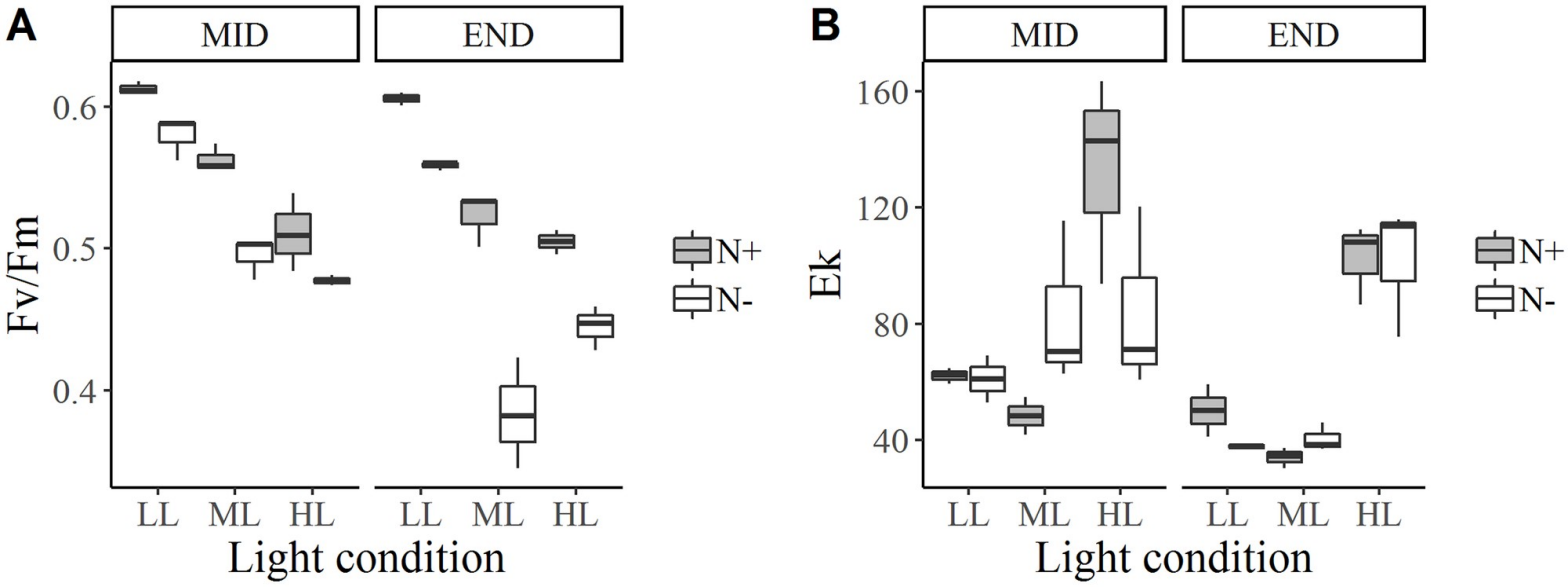
Box and whisker plots for *Staurosira* sp. photosynthetic parameters: Maximum PSII quantum efficiency, Fv/Fm (A) and maximum light saturation parameter, Ek (B) for each treatment and for two growth stages: Exponential growth phase (mid-growth) and stationary phase (end of growth).

For all species, Fv/Fm was higher in LL photoacclimated cultures (*p* < 0.001) and higher in N+ medium (*p* < 0.001) during mid-growth and at the end of growth (Figs 2–4). Fv/Fm was around 0.6 for all species at mid-growth, except for *Staurosira* sp. under HL, for where Fv/Fm was around 0.5 in N+ condition and 0.4 in N- condition (Fig 4).

For *E*. *paludosa* (Fig 2A), during mid-growth, Fv/Fm values were all superior to 0.58 ± 0.01. The same pattern was found for *N*. *alexandrina* (Fig 3A), where Fv/Fm values were mainly superior to 0.59 ± 0.01. These results indicate that cells were not stressed by the culture conditions during mid-growth, meaning that all major nutrients were available in the medium, even for *N*. *alexandrina* for which a limitation by phosphate was suggested (Table 3). For *Staurosira* sp. (Fig 4A) Fv/Fm values tend to decrease with light and with lack of nitrogen (*p* < 0.001). The highest Fv/Fm value was obtained under LL and N+ (0.61 ± 0.01) and lowest for HL and N- (0.47 ± 0.01).

In *E*. *paludosa*, at the end of the growth phase, Fv/Fm value was lower in N- than N+ in all light levels (*p* < 0.001); it dropped by 54.89 ± 4.78% for HL, 42.25 ± 3.84% for ML, and 22.38 ± 0.75% for LL. There was a significant interaction between light conditions and nitrogen concentration (*p* < 0.001). The stronger the light and lower the nitrogen concentration, the lower the Fv/Fm value became. As for growth, these results can be explained by nutrient consumption and demonstrate the effect of nitrogen limitation on cells.

For *N*. *alexandrina* (Fig 3A), Fv/Fm decreased between mid and end of growth for all treatments (*p* < 0.001) and Fv/Fm was higher under LL whatever the nitrogen concentration (*p* < 0.001), as for *E*. *paludosa*. However, no significant difference between N+ and N- was detected for HL or ML (*p* = 0.62). Even though cell concentration was higher under LL in N- (Fig 1), Fv/Fm value suggested that they were more stressed in N- than N+ conditions (*p* < 0.01). This can be explained by the accumulation of two nutrient limitations (nitrogen and phosphate) under N-, whereas only phosphate was limited under N+ (Table 3). These nutrient limitations can also explain why no difference between N+ and N- conditions was detected for ML or HL: nitrogen and phosphate limitations could possibly have the same effect on cells and trigger a stress.

For *Staurosira* sp. (Fig 4A), as for *E*. *paludosa*, Fv/Fm dropped between mid-growth and the end of growth, particularly under N- conditions (*p* < 0.001). The highest Fv/Fm value was found under LL whatever the nitrogen concentration (*p* < 0.001). These results can be explained by the nitrogen consumption inducing a stressful nitrogen limitation during growth.

The Ek parameter, which is an indicator of the state of cellular photoacclimation was, as expected, always higher under HL (*p* < 0.001) for all the species, showing that cultures were well photoacclimated. As a result of stress induced by nutrient consumption and growth, this parameter decreased between mid-growth and the end of growth (*p* < 0.001). This decrease could be induced by autoshading among cells, which implies a decrease in the brightness reaching them. Ek value was higher under HL for *Staurosira* sp. (133.3 ± 35.8) compared with *E*. *paludosa* (59 ± 3.6) and *N*. *alexandrina* (65.68 ± 10.74). These results can be explained by the fact that this latter species tends to be more in suspension in the medium than the other which increases the light reaching the cells.

### Pigments

**Fig 5:**
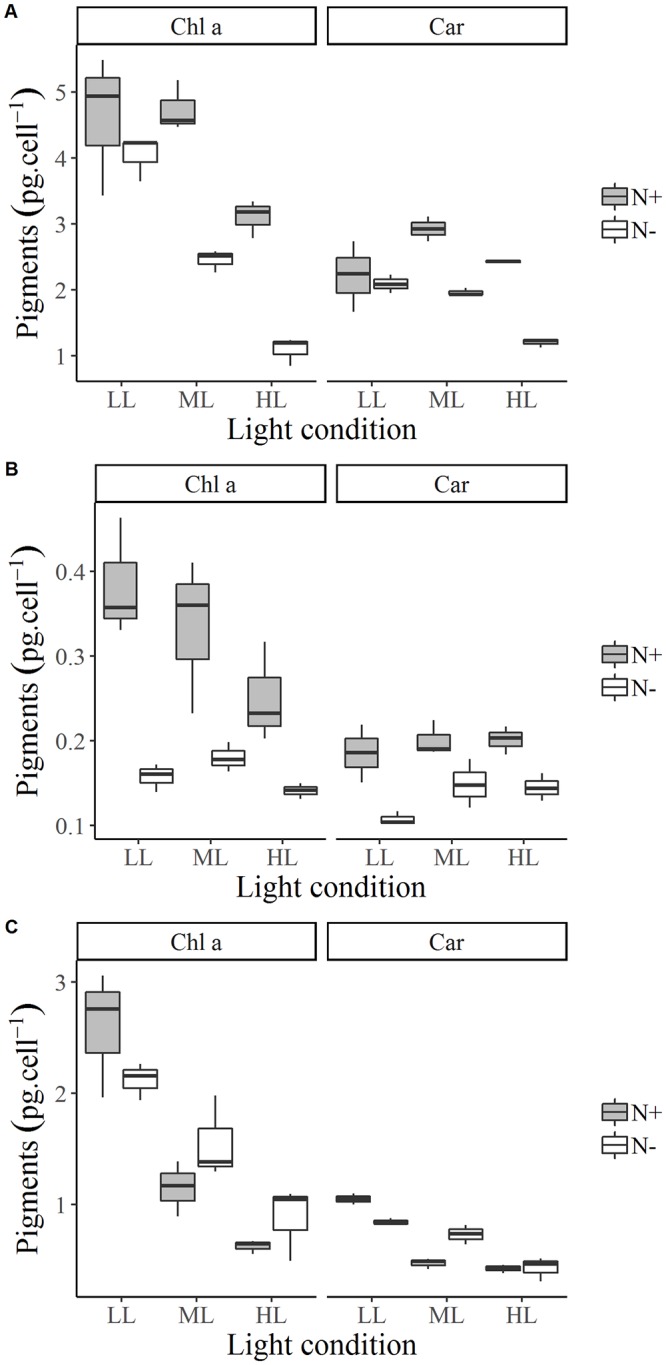
Box and whisker plots for *E*. *paludosa* (A), *N*. *alexandrina* (B), *and Staurosira sp*. (C) of Chl *a* content (pg.cells^-1^) and Carotenoid content (pg.cells^-1^) at the end of growth for each culture condition.

For *E*. *paludosa*, pigment concentrations, i.e., Chl *a* and Car, were impacted by both nitrogen (*p* < 0.001) and light conditions (*p* < 0.001) (Fig 5A). As expected, Chl *a* concentration in the cells was higher under LL (*p* < 0.01) due to their photoacclimation. Chl *a* concentration was lower in N-, especially for ML and HL (*p* < 0.001). Chl *a* content and Fv/Fm followed the same trend: decreasing Fv/Fm (Fig 2A) could be linked to a lower production of Chl *a* by stressed cells. Car concentration was lower in N- conditions (*p* < 0.001) and HL (*p* < 0.01), as for Chl *a*.

For *N*. *alexandrina*, pigment concentrations were affected by nitrogen (*p* < 0.001) but not by light levels (*p* = 0.073), although the expected decrease of Chl *a* with light can be observed (Fig 5B). Chl *a* and Car concentrations were higher under N+ than N- whatever light conditions (*p* < 0.001). As for *E*. *paludosa*, this tendency can be explained by nutrient limitation and contrasts with Fv/Fm results (which were similar between N+ and N-), as nitrogen limitation induced a halt or decrease in Chl *a* production. To our knowledge, crossed limitation has not been described in literature: nitrogen or phosphate limitation are addressed separately but neither together. However, on the basis of the current results, we hypothesize that the production of Chl *a* is mainly impacted by nitrogen limitation. Indeed, nitrogen limitation induces a decrease of RUBISCO which induces an accumulation of ROS leading to cell death and a decrease in Chl *a* content [51].

Phosphate limitation impacts the photosynthetic machinery in this strain (low Fv/Fm, Table 3), which can be explained by the involvement of phosphate in the synthesis of proteins, phospholipids and ATP that ensure the proper functioning of the photosynthetic apparatus [52].

For *Staurosira* sp. (Fig 5C), pigment concentrations were impacted by light (*p* < 0.001) but not by nitrogen (*p* = 0.70). As seen for *E*. *paludosa*, Chl *a* and Car concentrations were higher under LL (*p* < 0.001) whatever nitrogen concentration. The absence of differences in Chl *a* between N+ and N- can be explained by the low differences of nitrogen consumption between these two conditions. Thereby, for this species, Chl *a* concentration was more impacted by light level.

### Macromolecular content

**Fig 6:**
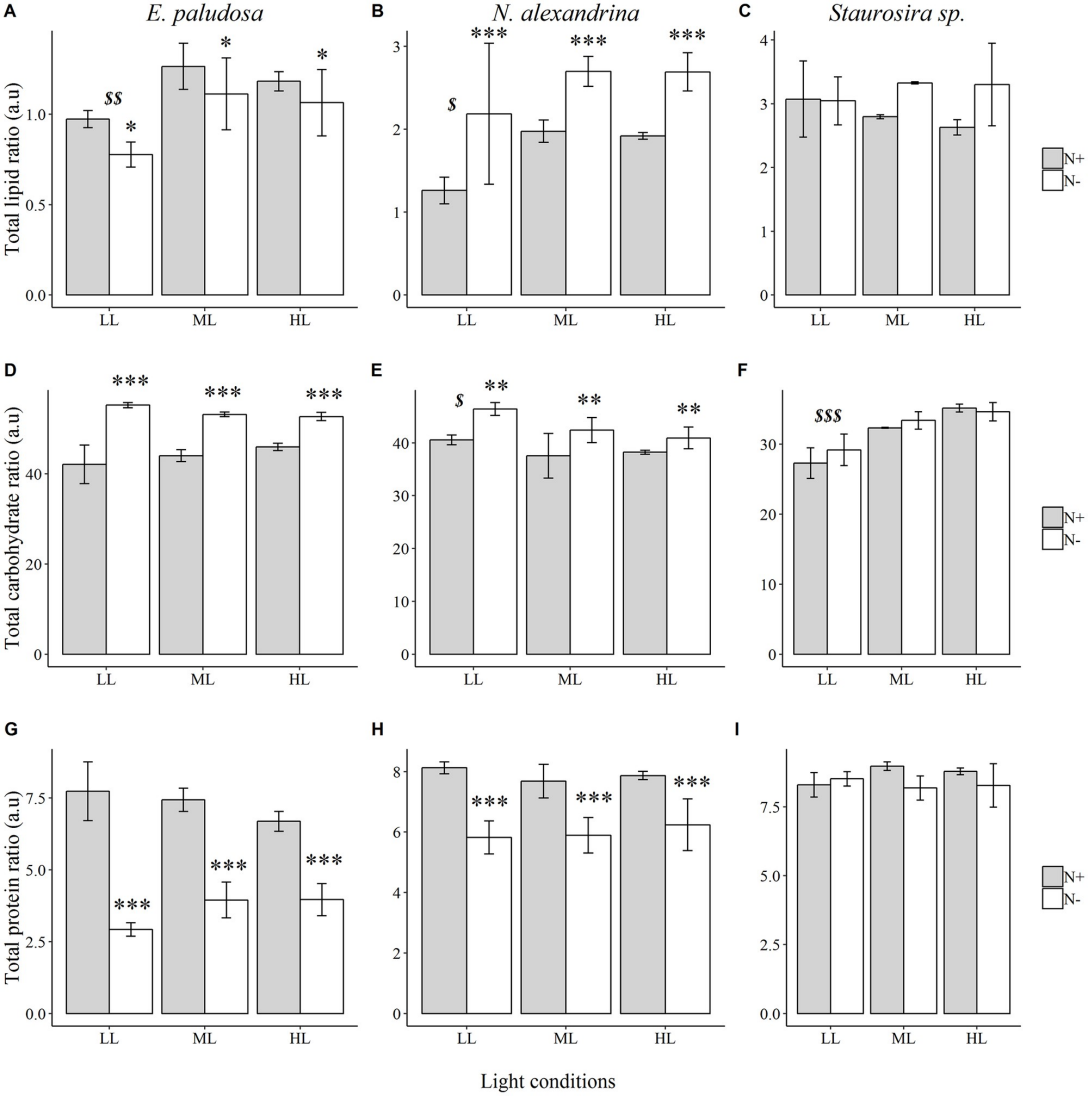
Total lipid (A,B,C), carbohydrate (D,E,F), and protein ratios (G,H,I) of *E*. *paludosa*, *N*. *alexandrina*, and *Staurosira* sp. for each treatment. * *p* < 0.05;**p < 0.01;***p < 0.001 for nitrogen conditions and ^$^ *p* < 0.05;^$ $^*p* < 0.01;^$ $ $^*p* < 0.001 for light conditions.

Macromolecular contents (lipids, proteins, and carbohydrates) measured using the FTIR method showed that *E*. *paludosa*, *N*. *alexandrina*, and *Staurosira* sp. have different biochemical profiles (Fig 6). Total lipid content decreased for *E*. *paludosa* grown in nitrogen limiting conditions (*p* < 0.05), and was also lower under LL (*p* < 0.05); however, it increased in *N*. *alexandrina* in the same nitrogen conditions (*p* < 0.001) but was still the lowest under LL (*p* < 0.05). Neither nitrogen (*p* = 0.06) nor light (*p* = 0.88) appeared to impact *Staurosira* sp. total lipid production (Fig 6A–6C). Carbohydrate content increased for both *E*. *paludosa* and *N*. *alexandrina* under nitrogen limitation (*p* < 0.001) (Fig 6D and 6E). However, carbohydrate concentration was not impacted by light level in *E*. *paludosa* (*p* = 0.84) but was higher under LL in *N*. *alexandrina* (*p* < 0.05). Protein synthesis for these two species was limited under N- conditions (*p* < 0.001) (Fig 6G and 6H). No impact of nitrogen concentration on carbohydrate and protein synthesis was found for *Staurosira* sp (Fig 6F and 6I) and the only effect observed for light was for carbohydrate production under LL (*p* < 0.001). Results for this species could be explained by the fact that the difference in nitrogen consumption between N+ and N- conditions was very small, indicating little limitation growth by nitrogen, which was also reflected by similar growth parameters (Table 4). *E*. *paludosa* and *N*. *alexandrina* cultured in stressful conditions remobilize carbon to produce energy storage products (carbohydrates and/or lipids) for acclimation to the altered nutrient conditions. For these two species, cells may produce less storage products (carbohydrates and proteins) when cultured under LL because they are less stressed, as already demonstrated by the high Fv/Fm.

### Biomass and lipid content

**Fig 7:**
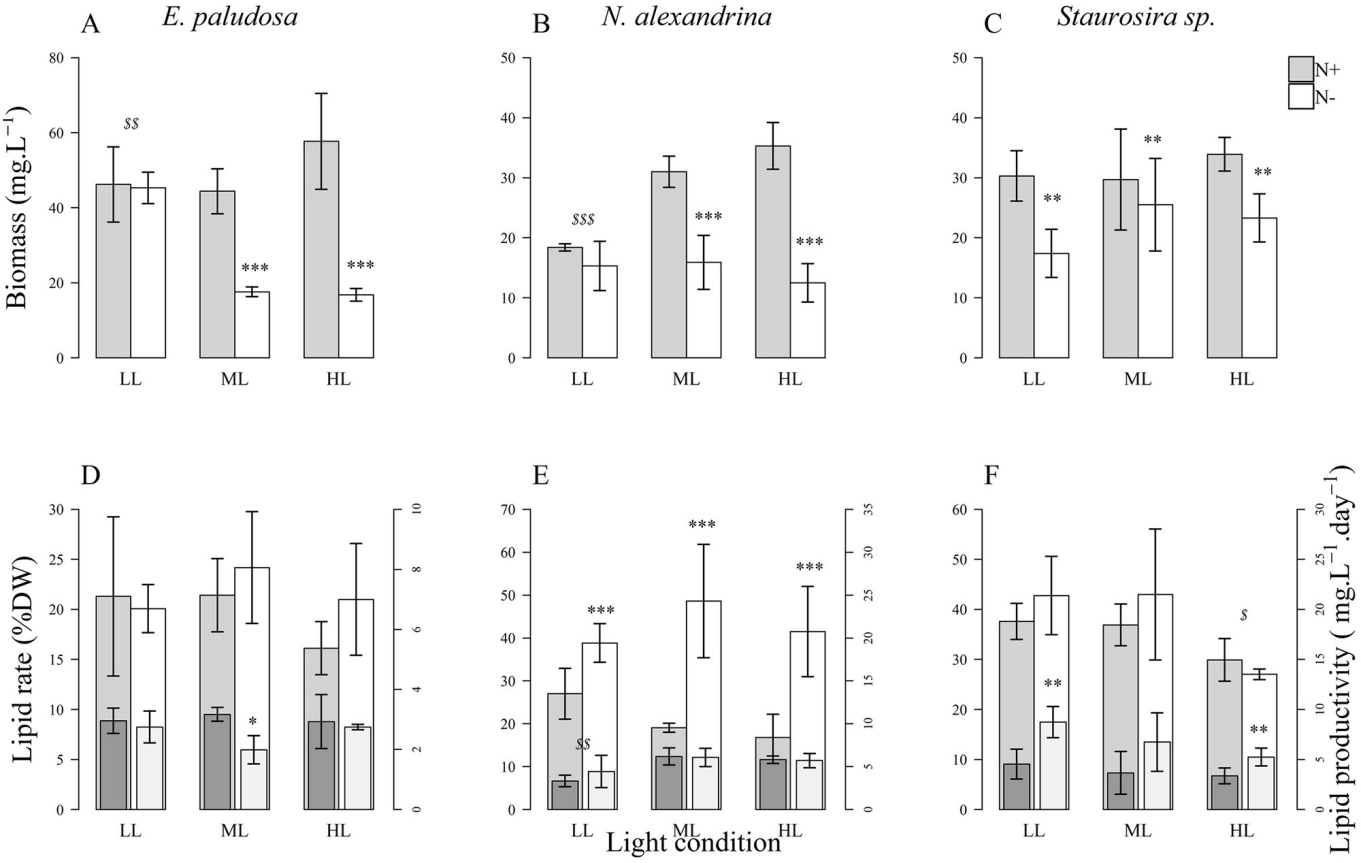
Biomass (A, B, C), lipid rate, and lipid production (D, E, F; high bar = lipid rate; low bar = lipid productivity) obtained at the end of the growth for *E*. *paludosa*, *N*. *alexandrina*, and *Staurosira sp*. for each treatment. * *p* < 0.05; ** *p* < 0.01; *** *p* < 0.001 for nitrogen conditions and ^$^ *p* < 0.05; ^$ $^ *p* < 0.01; ^$ $ $^ *p* < 0.001 for light conditions.

The highest biomass for *E*. *paludosa* (57.77 ± 12.86 mg.L^-1^), *N*. *alexandrina* (35.3 ± 3.9 mg.L^-1^), and *Staurosira* sp (33.9 ± 0.28 mg.L^-1^) was achieved under HL and N+ conditions (Fig 7A and 7C). For *E*. *paludosa* and *N*. *alexandrina* biomass was higher in N+ conditions than N- (*p* < 0.001) except for LL (*p* = 0.88). For *Staurosira* sp. biomass was higher in N+ conditions (*p* < 0.01) except for ML (*p* = 0.55). These results were directly linked to the growth parameters (Table 4, Fig 1).

Total lipid contents obtained by gravimetry where not impacted by light (*p* = 0.30) or nitrogen concentration (*p* = 0.79) in *E*. *paludosa* and corresponded to 20.68 ± 2.62% DW. However, lipid productivity was higher under N+ than N- conditions for ML (*p* < 0.05). For *N*. *alexandrina* total lipid content obtained by gravimetry was impacted by nitrogen concentration (*p* < 0.001) but not by light (*p* = 0.63). However, lipid productivity was the lowest under LL (*p* < 0.05). This confirmed previous observations: *N*. *alexandrina* accumulated lipids under nitrogen-limited conditions while *E*. *paludosa* did the opposite. Regarding photosynthetic parameters, *N*. *alexandrina* was less stressed under LL whatever the nitrogen concentration, explaining a lower lipid production under LL, whereas the highest lipid content for *N*. *alexandrina* was 42.99 ± 5.06%, obtained under stressful N- conditions. For *Staurosira* sp., total lipid content obtained with gravimetry was lower under HL (*p* < 0.05), but not impacted by nitrogen concentration (*p* = 0.41). Lipid productivity was higher under N- conditions (*p* < 0.01). Even though cells were stressed by nitrogen depletion (see Fv/Fm values Fig 4A), this apparently did not induce the production of lipids and/or carbohydrates as seen for the other two species. *Staurosira* sp. was the species with the highest lipid rate under replete nutrient conditions (36.21 ± 8.42%), whereas *N*. *alexandrina* was the only species that accumulated lipids under depleted nutrient conditions (42.99 ± 11.09%).

#### Fatty acid characteristics

The most abundant saturated fatty acid (SFA) was palmitic acid (C16:0), ranging from 18.1 ± 3.1% to 64.6 ± 6.0% of total FAs for all strains in all treatments. The most abundant unsaturated fatty acid (UFA) was oleic acid (C18:1) for *E*. *paludosa* and palmitoleic acid (C16:1) for *N*. *alexandrina* and *Staurosira* sp. (Table 5).

**Table 5:** FAME composition of *E*. *paludosa*, *N*. *alexandrina*, and *Staurosira* sp. for all treatments.

Fatty acid (% total fatty acid)	*E*. *paludosa*			*N*. *alexandrina*			*Staurosira* sp.		
	LLN+	MLN+	HLN+	LLN+	MLN+	HLN+	LLN+	MLN+	HLN+
C14 :0	10.0 ± 2.1	8.0 ± 5.4	10.1 ± 1.5	0.6 ± 1.1	1.5 ± 0.5	3.9 ± 1.9	1.9 ± 0.7	1.7 ± 0.8	1.3 ± 0.8
C15 :0				0.3 ± 0.4	0.5 ± 0.1	1.5 ± 1.1	0.5 ± 0.2	0.5 ± 0.2	ND
C16 :1	13.6 ± 4.1	11.8 ± 8.9	9.4 ± 2.6	12.7 ± 4.8	26.1 ± 2.9	22.6 ± 11.6	39.7 ± 14.0	35.1 ± 10.1	23.0 ± 4.6
C16 :0	31.7 ± 2.0	30.2 ± 1.3	36.5 ± 6.0	18.1 ± 3.1	23.6 ± 0.8	24.6 ± 6.4	37.7 ± 3.5	41.8 ± 2.4	47.4 ± 2.1
C16 :2 cj				ND	1.3 ± 0.5	2.9 ± 1.6	4.3 ± 5.2	4.6 ± 2.9	7.4 ± 3.8
C18 :2	1.0 ± 0.7	1.1 ± 1.0	0.3 ± 0.4						
C18 :1	19.2 ± 5.1	36.7 ± 23.2	8.9 ± 2.3	19.5 ± 15.1	9.4 ± 3.6	16.1 ± 14.6	5.5 ± 2.1	3.3 ± 0.4	2.8 ± 0.4
C18 :0	15.3 ± 5.0	5.2 ± 3.5	21.1 ± 4.7	11.1 ± 1.5	10.8 ± 4.4	7.7 ± 2.5	6.2 ± 3.0	9.0 ± 10.2	10.9 ± 6.7
C18 :02 cj				ND	ND	8.8 ± 10.3	0.8 ± 1.4	1.7 ± 2.7	3.8 ± 1.4
C20 :5 (EPA)				**1.5 ± 0.4**	**1.7 ± 0.2**	**0.3 ± 0.4**			
C20 :4 (ARA)				**10.8 ± 3.0**	**11.7 ± 3.1**	**2.3 ± 2.4**	1.1 ± 1.6	ND	ND
C20 :0				1.7 ± 0.5	0.9 ± 0.2	1.1 ± 0.5	0.5 ± 0.2	0.6 ± 0.2	0.8 ± 0.7
C22 :1				3.6 ± 1.2	3.7 ± 1.9	1.6 ± 1.8			
C22 :0				2.9 ± 1.4	0.8 ± 0.1	1.6 ± 1.2			
C24 :0				4.4 ± 1.8	1.7 ± 0.3	1.5 ± 0.2	0.5 ± 0.2	0.3 ± 0.3	1.0 ± 1.0
HC	9.3 ± 3.0	7.0 ± 6.6	13.7 ± 0.4	12.8 ± 5.7	7.8 ± 3.4	3.6 ± 2.9	2.1 ± 1.3	1.3 ± 1.0	0.5 ± 0.1
**UFA**	**33.6 ± 5.7**	**49.6 ± 15.2**	**18.6 ± 0.1**	**52.9 ± 4.9**	**52.6 ± 3.6**	**50.0 ± 8.7**	**51.4 ± 7.0**	**44.7 ± 14.0**	**37.0 ± 9.5**
MUFA	32.7 ± 4.8	48.5 ± 14.2	18.3 ± 0.3	40.8 ± 9.6	38.0 ± 2.0	39.5 ± 4.3	45.2 ± 11.9	38.4 ± 9.7	25.8 ± 4.4
PUFA	0.8 ± 0.9	1.1 ± 1.0	0.3 ± 0.4	12.2 ± 4.7	14.6 ± 2.8	10.5 ± 4.4	6.2 ± 5.7	6.3 ± 4.9	11.2 ± 5.1
**SFA**	**57.0 ± 2.6**	**43.4 ± 8.9**	**67.7 ± 0.3**	**37.4 ± 2.6**	**39.6 ± 4.8**	**45.0 ± 7.0**	**47.4 ± 6.8**	**53.8 ± 12.7**	**61.3 ± 7.6**
	LLN-	MLN-	HLN-	LLN-	MLN-	HLN-	LLN-	MLN-	HLN-
C14 :0	10.8 ± 2.7	9.9 ± 2.1	8.4 ± 2.4	4.6 ± 0.9	3.7 ± 1.0	3.0 ± 0.8	2.9 ± 1.2	3.7 ± 1.3	3.0 ± 1.1
C15 :0	0.9 ± 0.1	0.7 ± 0.1	1.1 ± 0.9	1.0 ± 0.2	1.0 ± 0.2	0.7 ± 0.1	0.3 ± 0.2	0.3 ± 0.3	0.7 ± 0.3
C16 :1	3.2 ± 0.2	4.0 ± 0.6	6.0 ± 2.1	25.2 ± 1.9	20.8 ± 3.6	25.2 ± 6.4	25.0 ± 9.0	6.9 ± 2.1	19.0 ± 7.0
C16 :0	36.6 ± 6.4	36.7 ± 6.9	33.3 ± 5.7	32.5 ± 4.3	39.2 ± 3.2	35.3 ± 1.2	50.9 ± 9.7	64.6 ± 6.0	58.0 ± 3.7
C16 :2 cj				5.5 ± 3.9	9.5 ± 2.0	5.8 ± 1.7	4.6 ± 3.8	9.5 ± 0.7	5.4 ± 2.3
C18 :2	1.4 ± 1.4	1.9 ± 1.8	0.9 ± 0.3						
C18 :1	11.3 ± 0.0	20.1 ± 3.2	12.8 ± 9.4	4.1 ± 0.6	5.7 ± 2.2	6.6 ± 2.8	4.6 ± 1.4	3.0 ± 2.0	3.9 ± 1.9
C18 :0	13.0 ± 4.0	13.5 ± 2.0	16.8 ± 4.2	8.9 ± 0.5	6.6 ± 0.9	13.0 ± 2.9	5.5 ± 0.9	4.6 ± 1.6	4.5 ± 0.5
C18 :02 cj				3.9 ± 1.9	4.7 ± 1.3	2.5 ± 0.7	ND	ND	0.3 ± 0.2
C20 :5 (EPA)				**1.0 ± 0.1**	**ND**	**ND**			
C20 :4 (ARA)				**3.9 ± 0.4**	**ND**	**ND**			
C20 :0	0.7 ± 0.3	1.2 ± 0.6	0.5 ± 0.5	0.8 ± 0.3	0.6 ± 0.2	0.8 ± 0.3	0.3 ± 0.2	0.2 ± 0.3	ND
C22 :1				1.7 ± 1.3	0.8 ± 0.3	1.3 ± 0.6			
C22 :0				0.7 ± 0.2	0.7 ± 0.1	0.7 ± 0.2			
C24 :0				0.9 ± 0.4	0.9 ± 0.1	1.0 ± 0.1			0.2 ± 0.2
HC	12.1 ± 1.9	18.1 ± 4.0	20.1 ± 8.8	3.3 ± 1.4	2.8 ± 0.9	5.0 ± 0.3	6.0 ± 1.0	7.2 ± 1.6	5.0 ± 1.0
**UFA**	**26.1 ± 13.8**	**26.1 ± 5.7**	**23.9 ± 6.2**	**43.9 ± 11.6**	**41.5 ± 1.2**	**40.5 ± 3.0**	**34.2 ± 9.3**	**19.4 ± 2.6**	**28.3 ± 6.4**
MUFA	25.2 ± 12.7	24.2 ± 3.8	23.2 ± 6.4	38.0 ± 8.4	27.4 ± 2.0	33.1 ± 3.9	29.6 ± 10.4	10.0 ± 1.9	23.0 ± 5.7
PUFA	0.9 ± 1.3	1.9 ± 1.8	0.7 ± 0.2	12.6 ± 4.2	14.1 ± 1.6	7.4 ± 3.2	4.6 ± 3.8	9.5 ± 0.7	5.4 ± 2.3
**SFA**	**61.7 ± 12.3**	**62.1 ±7.6**	**60.2 ± 4.9**	**46.7 ± 11.0**	**55.7 ± 2.0**	**54.5 ± 2.7**	**59.7 ± 8.4**	**73.4 ± 4.1**	**66.6 ± 5.5**

Cj: Conjugated, EPA: Eicosapentaenoic acid, ARA: Arachidonic acid, HC: hydrocarbons, UFA: unsaturated fatty acid, MUFA: Monounsaturated fatty acid, PUFA: Polyunsaturated fatty acid, SFA: Saturated fatty acid, ND: Not detected.

*N*. *alexandrina* was the only species that produced arachidonic acid (C20:4) and eicosapentaenoic acid (20:5). These fatty acids were mostly produced under N+ and their concentrations were higher under LL and ML compared with HL (*p* < 0.05). For *N*. *alexandrina* and *Staurosira* sp., under N-, UFA tended to decrease (*p* < 0.01) and SFA to increase (*p* < 0.001). *E*. *paludosa* produced a significant amount of hydrocarbons (9.81 ± 3.18–18.12 ± 4.02%), which increased under N- conditions as for *Staurosira* sp. (*p* < 0.01).

## Discussion

### Growth and photosynthetic performances

Compared with our previous study [6], the three species examined in the current article show lower growth and biomass. However, this difference could be explained by their previously estimated growth under more favorable conditions, in medium using enriched natural seawater and with continuous light. Continuous light is known to be favorable because diatoms grow faster under longer photoperiods [53]. The use of artificial seawater made it possible to control nutrient concentrations more precisely than in enriched natural seawater, but there can be shortages of essential elements in the basal salt mixture, omission of minor elements present in natural seawater, or contamination of reagent grade salts [54–56], any of which could explain the lower biomass attained in this study.

As expected, growth rate and biomass increase under ML and HL, especially under N+ [15,57]. Latency phase is mostly impacted by light condition and is higher under LL as previously seen in *Skeletonema costatum* [57], where latency phase was 5 days longer under LL (20 μmol.photon.m^-2^.s^-1^) than under HL (340 μmol.photon.m^-2^.s^-1^), or ML (100 μmol.photon.m^-2^.s^-1^). However, species specific responses occur. For example, growth rate was lower under N- conditions for *E*. *paludosa* while it was lower under LL for *N*. *alexandrina* and no difference was observed between all tested conditions for *Staurosira* sp. Nevertheless, the majority trends in the literature show that growth rate normally rises with light intensity up to a certain point where photoinhibition occurs [58–61]. In the present study, only *N*. *alexandrina* seems to follow this tendency while *E*. *paludosa* and *Staurosira* sp. seem to have higher resistance to light conditions in terms of growth. Growth patterns of *E*. *paludosa* and *N*. *alexandrina* showed that under light-limited conditions and whatever the nitrate concentration used, growth curves were similar. This similarity suggests that, at low light intensity, it is light rather than nitrogen availability that is growth limiting. Jauffrais et al. (2015) [62] found the same growth rate for *E*. *paludosa* when grown under N+ (1.15 ± 0.06 day^-1^) and N- (1.13 ± 0.04 day^-1^). However, growth rates found for this species in the present study are lower; this could be explained by artificial seawater composition in the medium, which differs slightly from this previous study. The artificial medium used by Jauffrais et al. contained more phosphate (72.4 μM) and H_3_BO_3_ (178 μM) and less Kbr (12.5 μM) and SrCl_2_ (37.5 μM).

Photosynthetic efficiency, estimated by the Fv/Fm parameter, decreased for all species during growth, especially under N- conditions compared with N+, except for *N*. *alexandrina*. For this latter species, the photosynthetic efficiency decreased similarly under N+ and N-. The general decrease can be explained by the deprivation of nitrogen, known to be the most important element contributing to the dry weight of microalgae cells [63,64]. The low value of the photosynthetic parameter for *N*. *alexandrina* under N+ could also be due to phosphate limitation [65]. Regarding the photoacclimation parameter Ek, as expected, lower values were obtained for LL acclimated cells due to their light harvesting complex modification to optimize light capture [66,67]. The variations in both Fv/Fm and Ek, according to light for the three species studied are consistent with Cruz et al. [68] in which the same trend was found for *Nitzschia palea* under HL (400 μmol.photons.m^-2^.s^-1^) and LL (20 μmol.photons.m^-2^.s^-1^) treatments. They obtained Ek values of 162.4 under HL and 44.3 under LL. In the present study, similar Ek values were found for *Staurosira* sp., with an Ek value of 133.36 ± 35.8 under HL and 62.16 ± 2.65 under LL. Ek values under LL for *E*. *paludosa* (43.06 ± 4.25) and *N*. *alexandrina* (48.6 ± 1.80) were similar to findings of Cruz et al. However, Ek values for these two species under HL were lower than *Staurosira* sp. Ek value under HL was 65.68 ± 10.74 for *E*. *paludosa* and 59 ± 3.6 for *N*. *alexandrina*. These results suggest that *N*. *palea* and *Staurosira* sp. have a better adaptation capacity to the stronger light than *E*. *paludosa* and *N*. *alexandrina*. Cruz et al. [68] found Fv/Fm value to be higher under LL (0.63) than HL (0.55) which is also in accordance with our study. In the same way, Jauffrais et al. (2016) concluded that Fv/Fm reflects photochemical processes dependent on chloroplast reactions that use ATP and reductants provided by photosynthesis. In our study, lower Fv/Fm values obtained under HL were associated with a lesser production of Chl *a*. It is proven that the decline in pigments due to HL exposure [68,69] or nutrient limitation [70,71] affect photosynthetic activity and thus impact Fv/Fm value.

Under nitrogen [24,29,64,72] and phosphorus limitation [32,73], a decline in cell pigment content often appears and induces chlorosis. This phenomenon leads to a decrease in photosynthetic efficiency as demonstrated in previous studies [70,74,75]. Among these, Zulu at al. (2018) [75] studied the photosynthetic machinery of the diatom *Phaeodactylum trinocornutum* upon exposure to nitrogen limitation. Degradation of this machinery was induced leading the cells to become chlorotic, the nitrogen pools to shut down and the cellular proteins to decrease. As a result, biomass production was negatively affected as in the current study. Even if cells are nitrogen limited, they continue to consume phosphate. To continue their growth, they must use intracellular inorganic storages, such as Rubisco (known to be N-rich), which stops pigment production [72].

Nutrient consumption is affected by culture conditions. Under replete conditions, nitrate and phosphate consumption are greater under HL and ML than under LL. This expected result is due to slower growth under LL where nutrient consumption is low. However, for *E*. *paludosa*, nitrogen and phosphate consumption were mainly affected by light conditions while for *N*. *alexandrina* and *Staurosira* sp., nutrient consumption was mainly affected by nitrogen concentration, whatever the light level. These results suggest that, in some species, increased irradiance results in higher nitrogen and phosphate consumption, as already shown in some other studies [76,77], while for other species, light conditions do not affect nutrient consumption: a relationship not currently described in the literature.

For *N*. *alexandrina*, it is important to specify that all the phosphate available in the medium gets consumed whatever the culture conditions in terms of light and nitrate. This similar phosphate consumption could be explained by the fact that several diatom species are able to store phosphate in excess in intracellular pools to be used in case of limitation [78]. This evolutionary advantage allowing the cells to cope with levels of environmental phosphate, which often is the first nutrient to be depleted [79] could also be present in *N*. *alexandrina*. with the objective of using microalgal strains in biotechnology, most studies applied light or nutrient stress without verifying the physiological parameter Fv/Fm [13,14,17,26,27,30,80]. If strains cannot bear the culture conditions, their macromolecular content may change. In our case, lipid quantity could be thereby increased, but not necessarily lipid quality. To obtain stable production of a valuable compound it is necessary to ensure the integrity of the photosynthetic machinery. The Ek parameter enables us to verify good cell acclimation and Fv/Fm enables us to confirm their physiological states. It is essential to find culture conditions that sustain good growth and physiological states, in our study these conditions are respected under LL and N+ conditions. Some studies took into account physiological parameters [12,16,58,64] but did not analyze the effects of different light intensity and nutrient limitation simultaneously or used different diatom strains from ours (*Pheodactylum tricornutum*, *Chaetoceros muelleri*, *Chlorella* sp. L1, *Monoraphidum dybowskii*, *Nannochloropsis oceanica*, *Thalassiosira pseudonana*, and *Dunaliella tertiolecta*).

### Macromolecular content

Microalgae cultured in stressful conditions remobilize carbon to produce energy storage products (carbohydrates and/or lipids) for later consumption to cope with the unfavorable conditions and to survive. Nonetheless, this accumulation mechanism is not well understood [81,82]. He et al., (2015) found a 30% decrease in lipid content for *Chlorella* sp. L1 and *Monoraphidium dybowskii* cultured under LL intensity (40 μmol.photons.m^-2^.s^-1^) compared with cultures grown under HL (400 μmol.photons.m^-2^.s^-1^) [58]. The same tendency occurs with the three strains analyzed in the present study, suggesting the same strategy in these diatom strains as in the chlorophytes studied by He et al.: under light starvation conditions (e.g., LL), limited energy is allocated to growth and, with the rising light intensity, more energy is supplied to the synthesis of storage materials (i.e., carbohydrates and lipids). In this study, whereas *E*. *paludosa* and *N*. *alexandrina* accumulated lipids under the highest light intensities, *Staurosira* sp. accumulated carbohydrates. However, these observations are not applicable to all diatom species. *N*. *alexandrina* shows a decrease in carbohydrate content with light intensity, as demonstrated previously by Vårum et al. [83] for the diatom *Skeletonema costatum*.

Nitrogen limitation also reduces the ability of microalgae to use carbon fixed during the photosynthetic process. This carbon is normally used for protein synthesis. However, the decline in protein synthesis does not prevent cells from storing energy, which is why a nitrogen deficit, may result in the accumulation of carbohydrates and/or lipids depending on the species [29,36]. Accumulation of carbohydrates under nitrogen limitation has already been reported for *E*. *paludosa* by Jauffrais et al., (2015) [62] with a carbohydrate content of 36% under nitrogen-replete conditions and 67% under nitrogen-limited conditions. To our knowledge, only one study exists for *Staurosira* sp. [84], showing low lipid accumulation under nitrogen depleted conditions: 36% under high N fertilization and 45% under low N fertilization. These results are not consistent with our study, however, probably due to the low difference in nitrogen consumption between N+ and N- conditions: the *Staurosira* strain used in our study did not seem to be stressed by the low nitrogen level tested and attained a high lipid content–over 25% of its DW and capable of exceeding 40%–regardless of the nitrogen and light conditions.

Fatty acid composition of *E*. *paludosa* and *Staurosira* sp. Were studied here for the first time whereas there are already a few studies concerning the *Nitzschia* genus [1,5,85,86] and several more concerning diatoms [2,3,5,16,57,85,87–90]. For example, Renaud et al., (1999) studied the gross chemical and fatty acid composition of 18 species including *Nitzschia* sp. [1]. Lipid content and fatty acid composition found for this diatom species are in accordance with our results, with a production of EPA and ARA occurring under 80 μmol.m^-2^.s^-1^ with a 12h/12h photoperiod in F/2 medium. Nitrogen limitation, seems to be the parameter with the greatest impact on the three strains analyzed. This limitation is especially efficient for lipid accumulation in *N*. *alexandrina* cultures. However, several detrimental effects of nitrogen limitation on photosynthesis were identified in our study and are in agreement with previous findings [29,70], such as a decrease in Chl *a* and a 10- to 40-fold protein content decrease, contributing to chlorosis [91]. A consequence of this lack of de novo protein synthesis is a decrease in acetyl CoA carboxylase activity, the first committed step in fatty acid biosynthesis [14,92]. Nitrogen limitation is truly stressful for the cells and can cause them to lose the ability to synthetize and accumulate qualitative fatty acids of economic interest [31]. In our study, *N*. *alexandrina* lost the ability to produce ARA and EPA when cultured under nitrogen limitation combined with ML or HL. This finding suggests that if the photosynthetic machinery is impacted, then lipid quality will also be. Moreover, in this study, when cells were stressed, they tended to produce more SFA but less UFA. This trend was previously observed by Yongmanitchai et al. (1991) on *Pheodactylum tricornutum* [17]. This species produces less EPA and tends to accumulate SFA (C16:0), but also some UFA (C16:1, C18:1), when it grows under nitrogen limitation. Xia et al. (2013) observed a similar trend in *Odontella aurita*, a species of interest as a feed in aquaculture, with a decrease in EPA (5.6 vs 2.2%) and ARA (12.9 vs 9.0%) under nitrogen limitation and an increase in SFA (25.4 vs 39.0%), which is in accordance with our study [93].

### Future prospects for the use of the strains

Diatoms are reported to have a higher lipid content than other algal classes [31]. A literature survey by Griffiths and Harrison of 55 microalgae species in various classes [94] showed that diatoms, as a class, have an average lipid content of 30.6%DW, while they found an average lipid content of 8.6%DW for Cyanobacteria, 23.17% DW for Chlorophyta, 20.5% for Ochrophyta and 23.8% for other classes (Dinophyta, Prasinophyta, Euglenozoa, and Haptophyta). To be considered as an efficient lipid producer in industrial terms, microalgae have to accumulate at least 20% of their dry biomass as lipids [95]. The three strains presented in this study exceed this threshold, making them good candidates for biotechnology applications: *E*. *paludosa* and *Staurosira* sp. could be used for biofuel production because of their high production of SFA and UFA. Fatty acids like EPA and ARA produced by *N*. *alexandrina* could find applications in the cosmetic or nutraceutical industries. The main conclusion of this study is in accordance with previous ones [12,16,58]: lipid quantity and quality depend fundamentally on culture conditions, including the light environment and medium composition. Control processing conditions for lipid production at industrial scale has a cost [31]. To be economically sustainable, chosen strains have to be consistent lipid producers under varied environmental conditions rather than strains with higher productivity but only under optimal conditions [31]. Implementation of lipid production processes for microalgae strains has to take into account these economic constraints. Knowing the physiological capacity of strains is a necessary requirement to optimize culture conditions. If strains cannot bear the culture conditions, which can be only assessed by physiological analysis, production of lipids of interest can be made impossible, as seen in this study under HL and N- conditions. If culture conditions are adapted to photosynthetic machinery, long-term lipid production can be assessed. From this point of view, even if nitrogen limitation and HL can enhance lipid content, the strategy would not be advisable for industrial-scale lipid production. For *E*. *paludosa*, nitrogen limitation is inefficient for increasing lipid production, especially under LL. Even though growth took longer, biomass was the same under HL or LL, and photosynthetic efficiency remained high. In terms of cost, the use of the LL is of particular interest since *E*. *paludosa* produces more UFA and SFA under LL and these conditions diminish the need for high power artificial light that otherwise increase the cost of microalgae production [96]

For large scale production, *N*. *alexandrina* should be grown under ML and N+ to have an high, biomass and preserved ARA and EPA production. If grown under higher light and N- medium this strain will lose its ability to produce ARA and EPA and physiological states will be affected which is not desirable for the long-term production of these compounds.

*Staurosira* sp produces high amounts of lipids under depleted and replete nutrient conditions, but UFA and, particularly, MUFA were preferentially produced under replete conditions while mostly SFA were produced under depleted conditions. This species can also be grown under LL as for *E*. *paludosa*. This species could be used for biodiesel production, which in agreement with the study by Huntley et al. [84]. This species should be grown under LL and nutrient-replete conditions to obtain equal proportions of MUFA and SFA compatible with biodiesel.

The three new strains studied here are good candidates for biotechnology applications due to the quantity and quality of their lipid production under simple culture conditions: LL and nitrogen enrichment. Indeed, using low light induces low light energy consumption and using a nitrogen depleted medium induces less maintenance since it’s not necessary to produce these species in a two-stage culture (nitrogen replete and nitrogen depleted). However, to increase lipid content and quality, it would be interesting to test other culture conditions, such as limiting other nutrients like phosphate for example, which is known to increase lipids content in diatoms without damaging photosynthetic machinery as nitrogen limitation does [32,97]. Development of a specific photobioreactor (PBR) [98–101] sustaining biofilm culturing could also be interesting because the three species are benthic and naturally form biofilms. The combination of high quantity and quality lipid production and with low water and light use by these species could represent an ideal solution for new development of sustainable economic activities.

## Conclusion

Effects of different nitrogen concentrations and light conditions on *Entomoneis paludosa*, *Nitzschia alexandrina* and *Staurosira* sp. was explored by the evaluation of growth, photosynthetic performance including photosynthetic efficiency, pigment content, macromolecular content (lipids, carbohydrates, proteins), and fatty acid composition. Nitrogen limitation stimulates the accumulation of carbohydrates for *Entomoneis paludosa* and the accumulation of lipids for *Nitzschia alexandrina*. An irradiance between 100 and 400 μmol.photons.m^-2^.s^-1^ stimulates the accumulation of lipids for *Entomoneis paludosa* and *Nitzschia alexandrina* while for *Staurosira* sp. it stimulates the accumulation of carbohydrates. Under HL and nitrogen-limited conditions, the content of proteins and pigments decline accompanying a decrease in photosynthetic efficiency. This finding supports our main conclusion that the development of a lipid production which must be a compromise between economic and ecophysiological constraints. The selection of strains that fulfill both aspects is the best solution for sustainable production of lipids at an industrial scale. In fact, an increase in lipid level does not mean an increase in the production of lipids of interests, indeed, while PUFAs are economically valuable, under light or nitrogen stress UFAs increase and PUFAs decrease with a halt in EPA and ARA production.

## Abbreviations

ARA: Arachidonic acid
CAR: Carotenoids
Chl *a*: Chlorophyll a
Cj: Conjugated
CLE: Crude lipid extract
DW: Dry weight
Eb: Ester bond
Ek: Photoacclimation parameter
EPA: Eicosapentaenoic acid
FA: Fatty acid
FAME: Fatty acid methyl ester
Fv/Fm: Maximum quantum efficiency
HL: High light
HTSXT-FTIR: Fourrier-transform infrared spectroscopy high-throughput screening extension
LL: Low light
LP: Lipid productivity
LR: Lipid rate
ML: Middle light
MUFA: Monounsaturated fatty acid
ND: Not detected
PAM: Pulse amplitude modulated
PSII: Photosystem II
PUFA: Polyunsaturated fatty acid
rETR: Relative electron transport rate
RLC: Rapid light curve
SFA: Saturated fatty acid
TAG: Triacylglycerol
UFA: Unsaturated fatty acid
μ: Specific growth rate
